# Semiconductor lasers with integrated metasurfaces for direct output beam modulation, enabled by innovative fabrication methods

**DOI:** 10.1515/nanoph-2022-0585

**Published:** 2023-01-12

**Authors:** Dandan Wen, Kenneth B. Crozier

**Affiliations:** Key Laboratory of Light Field Manipulation and Information Acquisition, Ministry of Industry and Information Technology, and Shaanxi Key Laboratory of Optical Information Technology, School of Physical Science and Technology, Northwestern Polytechnical University, Xi’an 710129, China; Department of Electrical and Electronic Engineering, School of Physics, and Australian Research Council (ARC) Centre of Excellence for Transformative Meta-Optical Systems, University of Melbourne, Melbourne, VIC 3010, Australia

**Keywords:** metasurface, nano-optics, plasmonics, semiconductor laser, vertical cavity surface-emitting laser (VCSEL)

## Abstract

Semiconductor lasers play critical roles in many different systems, ranging from optical communications to absorption spectroscopy for environmental monitoring. Despite numerous applications, many semiconductor lasers have problems such as significant beam divergence and polarization instability. External optical elements like objective lenses and polarizers are usually needed to address these issues. This Review will discuss how these issues have recently been dealt with by instead integrating metasurfaces into semiconductor lasers. This necessitates the development of innovative fabrication methods; these will also be the topic of this Review. Metasurfaces can be integrated on the emitting facet of a laser. This can help select the lasing mode or can be used just to modify the output beam properties without affecting the modes. They can also be integrated monolithically with lasers through waveguides, or work in an external cavity configuration. These integrated devices provide novel optical functions, such as direct orbital angular momentum (OAM) mode generation, wavelength tuning and holographic pattern generation. We hope this Review will help extend the use of metasurface-integrated semiconductor lasers to scientific and industrial systems that employ lasers.

## 1 Introduction

Semiconductor lasers have become ubiquitous in everyday life, with vertical cavity surface emitting lasers (VCSELs) for example being used in smartphones for facial recognition and other laser types being used in laser printers. Like all lasers, a semiconductor laser consists of a gain mechanism and a cavity [1]. The gain is achieved by stimulated emission, generally by the recombination of holes and electrons via a forward biased p–n junction or via optical pumping. The semiconductor material must be a direct-bandgap type to generate the gain needed for lasing. Cladding layers typically surround the active region to form a waveguide structure, where the guided modes are confined in the active region. The cavity can be created by cleaving or etching the structure’s end for an edge-emitting laser, or by distributed Bragg reflectors for a VCSEL. Semiconductor lasers usually have small volumes, long operating lifetimes and high modulation frequency. Their operating voltage and current are compatible with integrated circuits, which facilitate monolithic integration. Despite numerous benefits, semiconductor lasers still have shortcomings. For instance, the polarization of the beam from a VCSEL is usually not stable since there is no robust mechanism for polarization selection [2]. Furthermore, the beam from a semiconductor laser usually has significant divergence (especially for an edge emitting laser), which makes it difficult to couple into a waveguide without external collimating optical elements.

Examples of non-metasurface techniques to control the output beams of semiconductor lasers have included monolithically-integrated diffractive optical elements (DOEs), micro lenses, and micro electro mechanical systems (MEMS). Monolithically-integrated DOEs can be divided into two categories, namely those produced by etching of the semiconductor and those produced by adding a structure to the device surface [3]. One of the earliest work of the former type by Rastani et al. [4] in 1991. A Fresnel lens was etched into the substrate of a bottom-emitting VCSEL with selective ion-beam milling. The output beam can be focused, collimated, and bent using such lenses. Similar technique can be used to make Fresnel lens on the top-emitting surface of a VCSEL, which works as a wavefront shaper and electric contact simultaneously [5]. In comparison with binary DOEs like Fresnel lenses, special processing methods, such as anisotropic chemical etching [6], are needed to directly machine microlenses with convex or concave shapes into III–V semiconductor materials. An alternative way of defining micro/nano structures on semiconductor lasers is to add a polymer layer to the output facet and then to pattern or shape it. The latter can be done with techniques that include thermal resist reflow [7], contactless hot embossing [8], thermally curving [9], and so on [10, 11]. The combination of semiconductor lasers with MEMS presents a powerful opportunity for dynamic optical beam control. Novel applications such as gas sensing [12], ophthalmic imaging [13] and LiDAR [14] have been demonstrated.

Enabled by the development of nanofabrication techniques, metasurfaces can be used to change the properties of light in a more flexible way in comparison with DOEs. Metasurfaces are composed of a single layer [15] or a few layers [16] of subwavelength nanostructures, and can be used to manipulate light beams. In the last decade, metasurfaces have drawn much attention due to the following benefits [17–20]: First, metasurfaces diminish our dependence on the need to use propagation to achieve the appropriate phase delay needed in an optical device, which opens up possibilities for ultrathin optical devices [21–23]. Second, metasurfaces can be used to achieve novel functionalities that do not exist in natural materials, such as negative refraction [24, 25], the giant spin-Hall effect [26–29] and general Pancharatnam–Berry Phases [30]. Finally, metasurfaces are more straightforward to fabricate than 3D metamaterials [31, 32]. Recently, there has been much interest in integrating metasurfaces with semiconductor lasers to form integrated systems. This leads to challenges both in design and fabrication. Here, we will briefly review recent works in this field. We focus on the operating principles (i.e. design) and the fabrication techniques. This Review is organized as follows: In Section 2, we describe semiconductor lasers with on-facet metasurfaces. We start from plasmonic nanostructures that are defined on the laser facet for beam collimation and polarization control. We then describe devices with a metasurface on the laser facet designed to achieve more complicated functionalities such as OAM generation. After that, we discuss VCSELs in which the cavity mirrors are high contrast gratings. In Section 3, we describe lasers monolithically integrated with metasurfaces through waveguides. In Section 4, we discuss external cavity semiconductor lasers incorporating metasurfaces. Here, the metasurfaces either provide feedback (for lasing) or work as active devices, i.e. generate light themselves. In Section 5, we conclude the paper with our thoughts on the future development of integrated devices.

## 2 Semiconductor lasers with on-facet metasurfaces

Integrating a metasurface on the laser facet might well be the most straightforward way to take advantage of both. Depending on the design, the metasurface could interact with the laser in two ways. First, the metasurface might have minimal effect on the lasing modes in the cavity and only modifies the output beam’s properties. Second, the metasurface could help select the lasing mode. In this section we will review three different configurations. These control the output beam’s wavefront, polarization, and frequency.

### 2.1 Beaming effect with slit-grating structure

**Figure 1:**
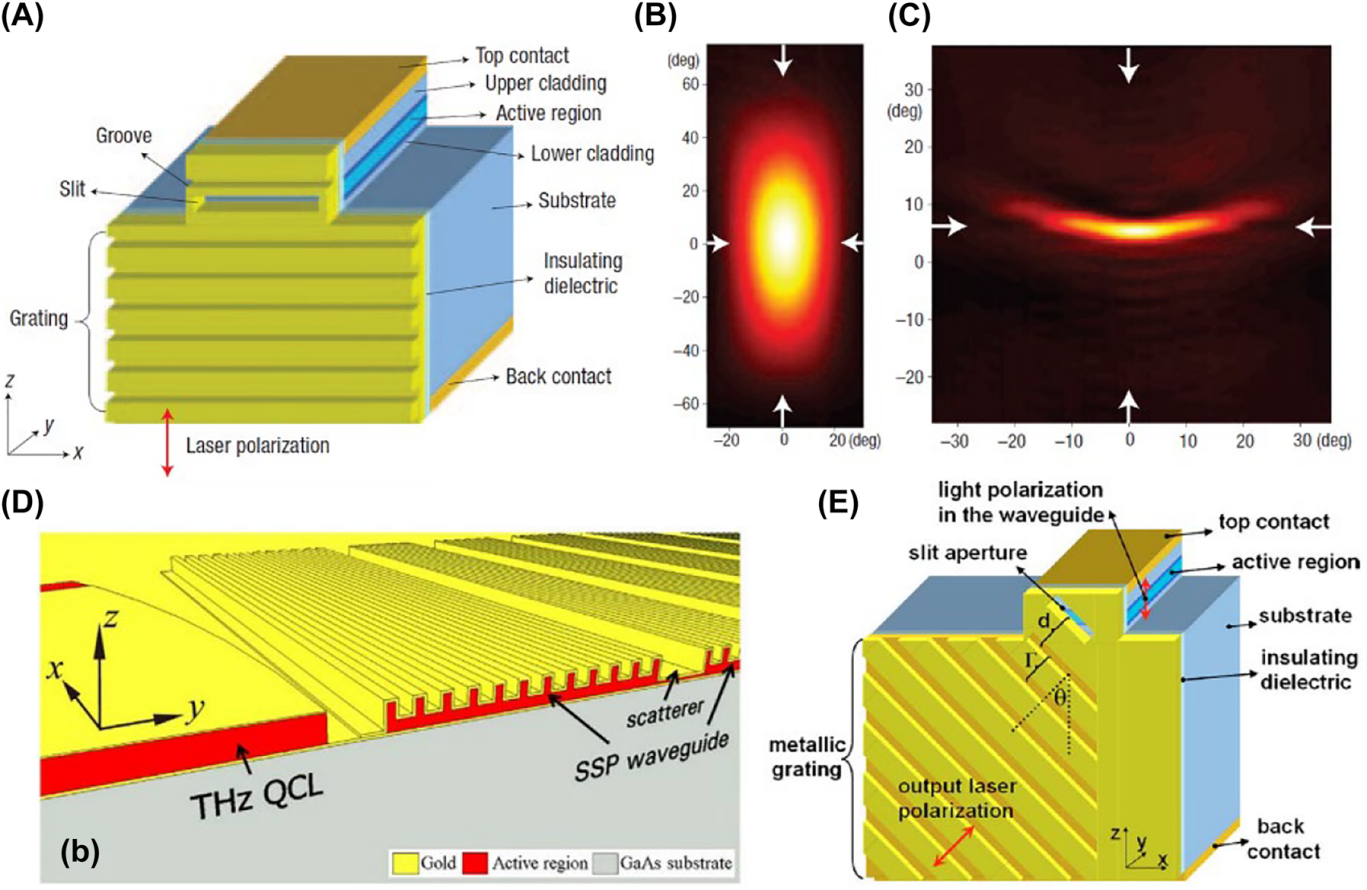
Laser collimation and polarization control with beaming effect. (A) Schematic of the QCL with slit-grating structure. (B–C) Experimentally obtained far-field intensity distributions when the QCL is without/with the slit-grating structure. It can be seen that the divergence is significantly compressed in the vertical direction after patterning the nanostructures. (D) Schematic of the THz QCL and the integrated metasurface, which contains a spoof surface plasmon waveguide and scatterers. (E) Schematic of a plasmonic polarizer on the laser facet, which consists of a slit aperture and metallic grating. Reprint permission obtained for references [33, 36, 37].

Edge-emitting semiconductor lasers, e.g. quantum cascade lasers (QCLs), usually have significant beam divergence due to the limited aperture size. However, for many important applications, such as coupling light into waveguides, it is important that the beams have only moderate divergence. Traditional methods for collimating laser beams usually involve external optical elements (lenses, etc.) and precise alignment. Yu et al. [33] defined a slit-grating structure on the facet of the QCL to reduce the divergence of the output beam (Figure 1A). The grating grooves are first etched on the facet of the bare substrate by a focused ion beam (FIB). Alumina and gold thin films are then deposited with electron beam evaporation. Here, the alumina serves as an electrical insulator. A two-angle-deposition procedure is used to ensure the coverage of the alumina-gold film on the side walls of the grooves. Finally, an elongated slit is opened with FIB on the emitting surface of the QCL. The laser beam (inherently polarized along the *z*-direction) is coupled to surface plasmons (SPs) through the slit and propagates along the grating. The SPs are coherently scattered by the grating into the far-field, leading to a decreased divergence angle (Figure 1B–C). This has been termed the “beaming” effect [34, 35].

The grating structure in Figure 1A can be modified to contain two separate regions. One region is close to the slit, and the other is far from it. They have different periods, providing different reciprocal lattice vectors to the surface plasmons. Thus, the outgoing laser beam will be diffracted in two directions [38]. Similarly, suppose we have a special QCL consisting of two stacks of active regions and emitting two wavelengths in the same direction. In that case, they can be spatially separated by the single-period structure similar to Figure 1A [38]. One can also use two-dimensional aperture gratings, instead of one-dimensional gratings, so that the laser divergence can be controlled in two orthogonal directions [39]. This approach also works well at other frequency bands. As shown in Figure 1D, a terahertz QCL can transform its beam to spoof surface plasmon waves, which propagate in the grating-type waveguide and are outcoupled by the scatterers to form a collimated beam in the far field [36].

The beaming effect has another interesting application, namely that of polarization filtering [37]. As shown in Figure 1E, by appropriate choice of the geometrical parameters of the slit aperture, one can ensure that only the light polarized perpendicular to the slit (along *θ* direction) is coupled into SPs propagating along the grating. The light beamed by the grating and most direct emission from the slit are polarized in the *θ* direction. These beams interfere to make the output *θ*-polarized. A circularly polarized beam is also possible, obtained by patterning two slit-grating structures on the laser facet. The two structures generate beams polarized along *θ*- and *θ* + *π*/2 directions and with a phase difference of *π*/2 between them. As a result, the nanostructures on the facet function like a quarter-waveplate, thereby enabling circular polarization generation.

### 2.2 Metasurfaces on the laser emitting facet

In addition to beam collimation, more complicated wavefront shaping functions have been demonstrated, among which the direction generation of OAM modes from a semiconductor laser is an important topic. A paraxial optical beam with helical wavefront exp(i*lϕ*) carries intrinsic orbital angular momentum, where *l* and *ϕ* are the topological charge and azimuth angle, respectively [28]. OAM beams have attracted much research interest and have been applied in optical communications [42, 43], imaging [44] and micromanipulation [45]. Usually, we need external elements such as q-plates [46], fork gratings [47] and metasurfaces [48–53] to change a Gaussian beam into an OAM beam. By inserting optical elements such as q-plates [54] and metasurfaces [55] into a solid-state laser cavity, light beams with pure OAM states and superposed OAM states can be directly generated from a laser. While exciting results have been obtained, ideally one would have a more integrated solution. Here we examine this, limiting our discussions to integrating metasurfaces on the facets of semiconductor lasers for OAM mode generation.

**Figure 2:**
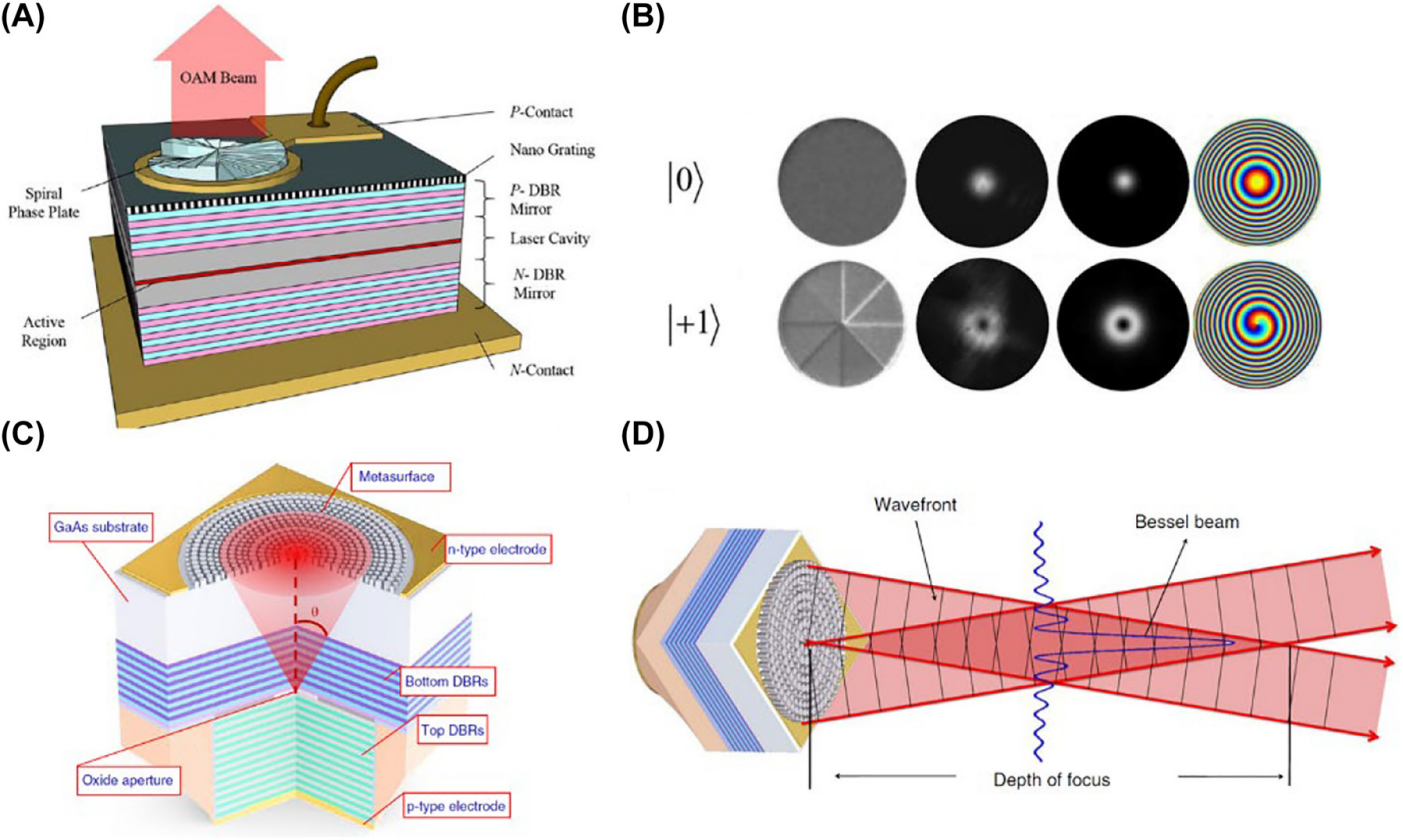
Metasurfaces on the laser facet for wavefront control. (A) Schematic of the VCSEL with an integrated spiral phase plate on the emitting surface for direct OAM beam generation. (B) First column: Scanning electron microscope (SEM) image of the fabricated spiral phase plate; second and third columns: experimentally obtained and simulated intensity profiles of the generated OAM beams, respectively; fourth column: simulated phase patterns of the OAM beam. The top and bottom rows represent the case for *l* = 0 and *l* = 1, respectively. (C) Schematic of metasurface integrated on back-side emitting VCSEL. (D) Schematic of VCSEL for Bessel beam generation. Reprint permission obtained for references [40, 41].

Li et al. [40] defined a micro-scale spiral phase plate on the emitting surface of a VCSEL for OAM mode generation and superposition. The phase term of an OAM mode is imparted via a silicon nitride phase plate with a gradient in its thickness around the plate center. As shown in Figure 2A, the phase plate thickness varies from zero to full height (corresponding to the phase change of 0–2*π*) and cycles three times in one revolution. This means that the generated OAM mode has a topological charge of three. The dielectric phase plate modulates wavefronts differently from metasurfaces with subwavelength scatterers. We nonetheless discuss it in this Review because we believe that its working mechanism and integration process may inspire future work on metasurface-based devices. To fabricate the device, the following is performed. A silicon nitride layer (1 μm thick) is first deposited on the top surface of the VCSEL with plasma-enhanced chemical vapor deposition. Photoresist is then used to protect the emitting surface of the VCSEL, followed by reactive ion etching (RIE) to remove the silicon nitride (except for the protected area). To form the phase plate, FIB is then used to define multilevel steps in the Si_3_N_4_ film. The simulated and experimentally obtained intensity patterns of the OAM beams are shown in Figure 2B. In addition to single OAM generation, OAM superposition can be achieved via a concentric design where the center and outer areas have different topological charges. In the fabrication process, the center of the plate must be well aligned with the center of the emitting surface to prevent the desired OAM mode from leaking to neighbouring modes [56].

Distributed feedback (DFB) lasers play a critical role in high-capacity optical fibre communications and in emerging applications such as fibre sensing, gas sensing and disease diagnosis. A DFB laser has a periodic structure in the laser gain medium, such as distributed Bragg grating. Stellinga et al. [57] use an Archimedean spiral grating to provide feedback and to control the phase and polarization of the output beam. Their device is a vertically-emitting DFB organic laser. Nanoimprint lithography is used. A silicon spiral grating is first fabricated with e-beam lithography and RIE. It is then used to cast daughter stamps in an elastomeric polymer. The daughter stamps transfer the spiral grating pattern into a UV-curable resist. Finally, the organic semiconductor BBEHP-PPV is spin-coated on the resist grating to form the device. Azimuthally polarized vortex beams with topological charges up to three are experimentally demonstrated.

Since VCSELs have small emitting surfaces and mesa-type cavities, direct fabrication of high-aspect-ratio nanostructures on the emitting surfaces is not straightforward. To address this challenge, Xie et al. [41] fabricate nanopillars on the substrate of their back-emitting VCSEL (Figure 2C). The method has two significant advantages. First, the GaAs substrate is suitable for the metasurface, which requires high transmittance and 0–2*π* phase modulation capacity. Second, the substrate surface is flat, facilitating the use of standard e-beam lithography and dry etching. The generation of a zero-order Bessel beam (Figure 2D) and the collimation and steering of beams are demonstrated. This technique can be used for applications of wavefront control, such as the multichannel generation of OAM beams with spatially varying topological numbers [58] and the generation of holographic images with a large field of view [59]. It is also demonstrated that a 2D Dammann grating can be fabricated on the substrate of a bottom-emitting VCSEL [60] so that multiple equal-power diffraction orders are generated.

**Figure 3:**
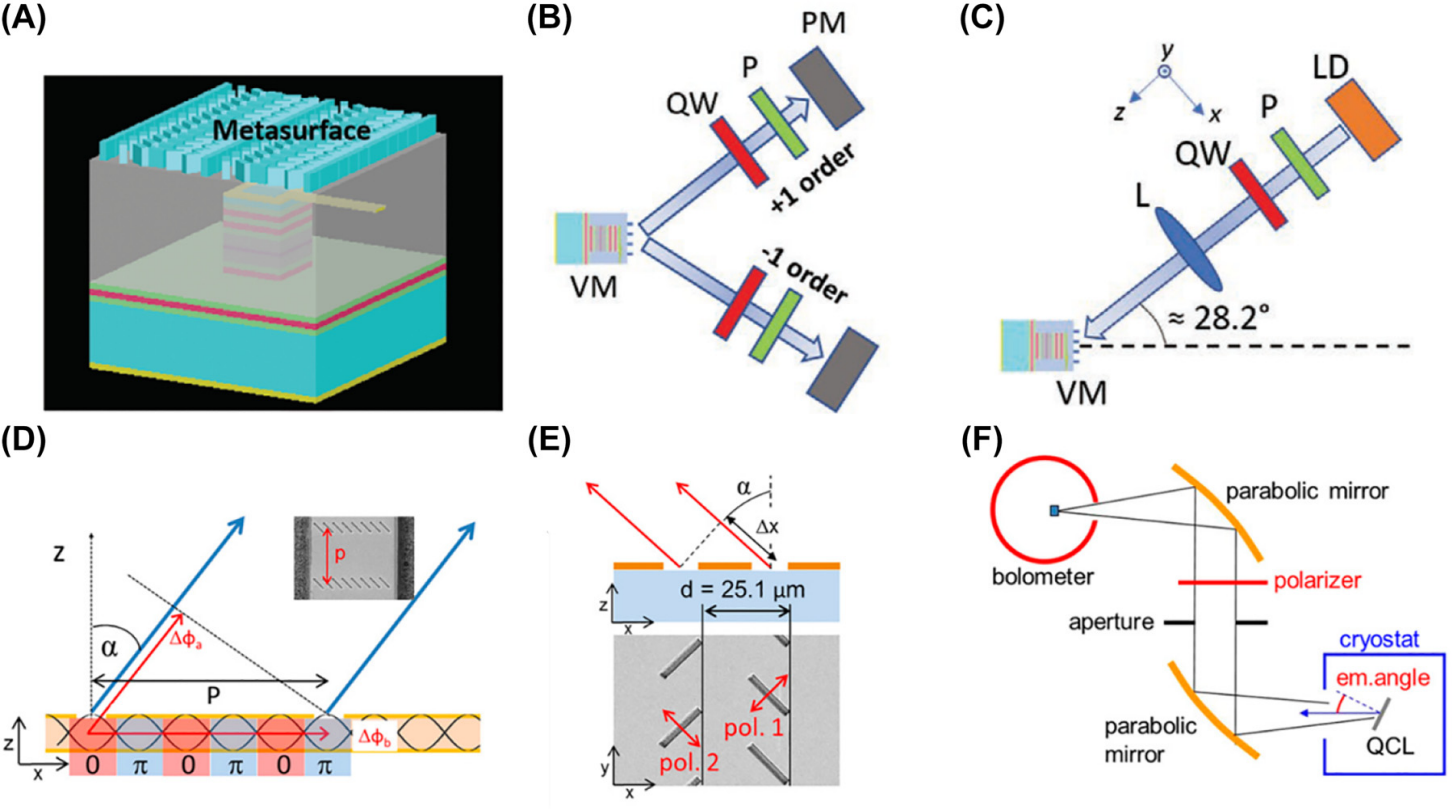
Metasurfaces on the laser facet for polarization control. (A) Schematic of the amorphous silicon metasurface integrated with VCSEL. (B) Experimental setup to verify the polarization states of the ±1st orders. VM: VCSEL with metasurface. QW: quarter wave plate. P: linear polarizer. If we properly choose the transmission direction of the polarizer and the fast axis direction of the quarter wave plate, the LCP and RCP components of the ±1st orders diffracted light can be separately measured. These can then be used to calculate the degree of circular polarization. (C) Experimental setup to verify the handedness of the incident circularly polarized light. L: lens. Light from a laser diode module illuminates the device along the opposite direction of the first diffraction order. The transmission axis of the polarizer is along the *y*-direction. We rotate the quarter-wave plate to generate LCP and RCP light. A lens is used to loosely focus the beam onto the VCSEL. (D) Schematic of the fifth-order grating on the top metallization of the metal-metal waveguide structure QCL. p: grating period. α: angle at which the beams interfere constructively in the far field. Δ*ϕ*_a_: phase shift of the neighbouring apertures caused by the beam propagation. Inset: SEM image of the grating structure. (E) Illustration of the phase shift between the two types of nanoapertures caused by the path difference Δ*x*. The device aims to generate elliptical polarization. (F) Experimental setup for the device characterization. The emitted light from the QCL is filtered by a polyethylene wire-grid polarizer between two parabolic mirrors and recorded by a bolometer. The polarizer is rotated and the recorded power is used to characterize the polarization of the emitted light. Reprint permission obtained from references [61, 62].

Another fabrication method to integrate metasurfaces with VCSELs was recently proposed by Wen et al. [61]. As shown in Figure 3A, the mesa-structure of VCSEL is embedded in a transparent polymer that serves as a platform for metasurface integration. The fabrication procedures are as follows: A Ti/Au hollow square electrode is first fabricated on the p-side of the VCSEL wafer with e-beam lithography and the lift-off process. The p-electrode is protected by photoresist (AZ4562), and dry etching is then performed to form the VCSEL mesa structure. The photoresist is removed, and another layer of photoresist (SU-8) is spin-coated over the wafer to embed the mesa fully. Dry etching is then used to remove the top SU-8 and expose the p-electrode. This is followed by the fabrication of a metallic pad and connecting wires on the SU-8 layer. Another SU-8 layer is then spin-coated to planarize the device and provide a platform for metasurface integration. Two types of metasurfaces are fabricated, to manipulate circular and linear polarizations. The first consists of amorphous silicon nanopillars that provide opposite phase gradients to left- and right-circularly polarized (LCP and RCP) incident waves. Thus, the two orthogonal circular polarized components are separated and sent to different directions (Figure 3B). It is also shown that the device can be operated as a detector. This is done by reverse biasing it, while illuminating it with light in the first-diffraction-order. It is shown that the resultant photocurrent depends on the helicity of the incident light (Figure 3C). The second type of metasurface, a bilayer grating, is also integrated on the VCSEL. It generates linearly polarized light when positive biased, and distinguishes between *x*-/*y*-polarized light when reverse biased (i.e. operating as a detector). The method provides a general solution to integrate metasurfaces on different semiconductor lasers with finite emitting surface areas and mesa structures. Wang et al. [50] fabricated a metasurface and a laser diode separately to simplify the fabrication procedure. The GaN nanograting metasurface functions as a quarter-wave plate. It is manually integrated on the emission window of the laser diode to transform the linearly polarized emission from the diode into circularly polarized light.

In Figure 1E, the slit aperture functions as a polarizer, which couples the light polarized perpendicular to its long axis into SPs. Here we will show that a metasurface consisting of a slit aperture array can also be used for polarization control. Rauter et al. [62] describe a surface-emitting THz QCL with a metal-metal waveguide structure. An aperture antenna array is etched into the top copper metallization by FIB milling. Each aperture functions as a polarizer that only scatters light polarized perpendicular to its length (Figure 3D). The apertures here are arranged in a fifth-order grating configuration that enables directional scattering. The grating period *p* equals *mλ*_0_/(2*n*_eff_), where *m* is the grating order (*m* = 5 here), *λ*_0_ is the wavelength in vacuum and *n*_eff_ is the effective refractive index of the mode in the waveguide. As a result, arbitrary linear polarizations can be achieved by controlling the orientation of the nanoapertures. Two types of apertures of orthogonal direction can be arranged with a displacement of half of the grating period, which causes a phase difference of *π*/2 when they interfere in the far-field (Figure 3E). Due to the standing wave pattern in the cavity, the two apertures are usually located at positions of different intensities, which makes the two apertures emit light of different powers. Thus, the resultant emission is elliptically polarized, rather than circularly polarized. The experimental setup used to characterize this device is shown in Figure 3F.

The metasurfaces mentioned above do not provide sufficient feedback to affect mode-selection. On the other hand, if the feedback is strong enough, it can be used to control mode selection, which could be beneficial for many applications, such as stabilizing the polarization state of a VCSEL. Mukaihara et al. [63] fabricated an Au-SiO_2_ grating on the cap layer of a VCSEL, to serve as a birefringent element for polarization control. A SiO_2_ grating is first fabricated on the cap layer with e-beam lithography and dry etching. After that, a layer of Au is deposited on the grating to bury the grooves. The phase of the light reflected from the grating differs for the two orthogonal linear polarizations. This leads to one polarization experiencing an antiresonance condition while the other is in a resonant condition. The resonant one has a higher gain, and lasing thus occurs in this mode. Mode selection can also be achieved by defining gratings on the top distributed Bragg reflector (DBR) [64] or on the cap layer [65].

### 2.3 High contrast grating for VCSEL property control

**Figure 4:**
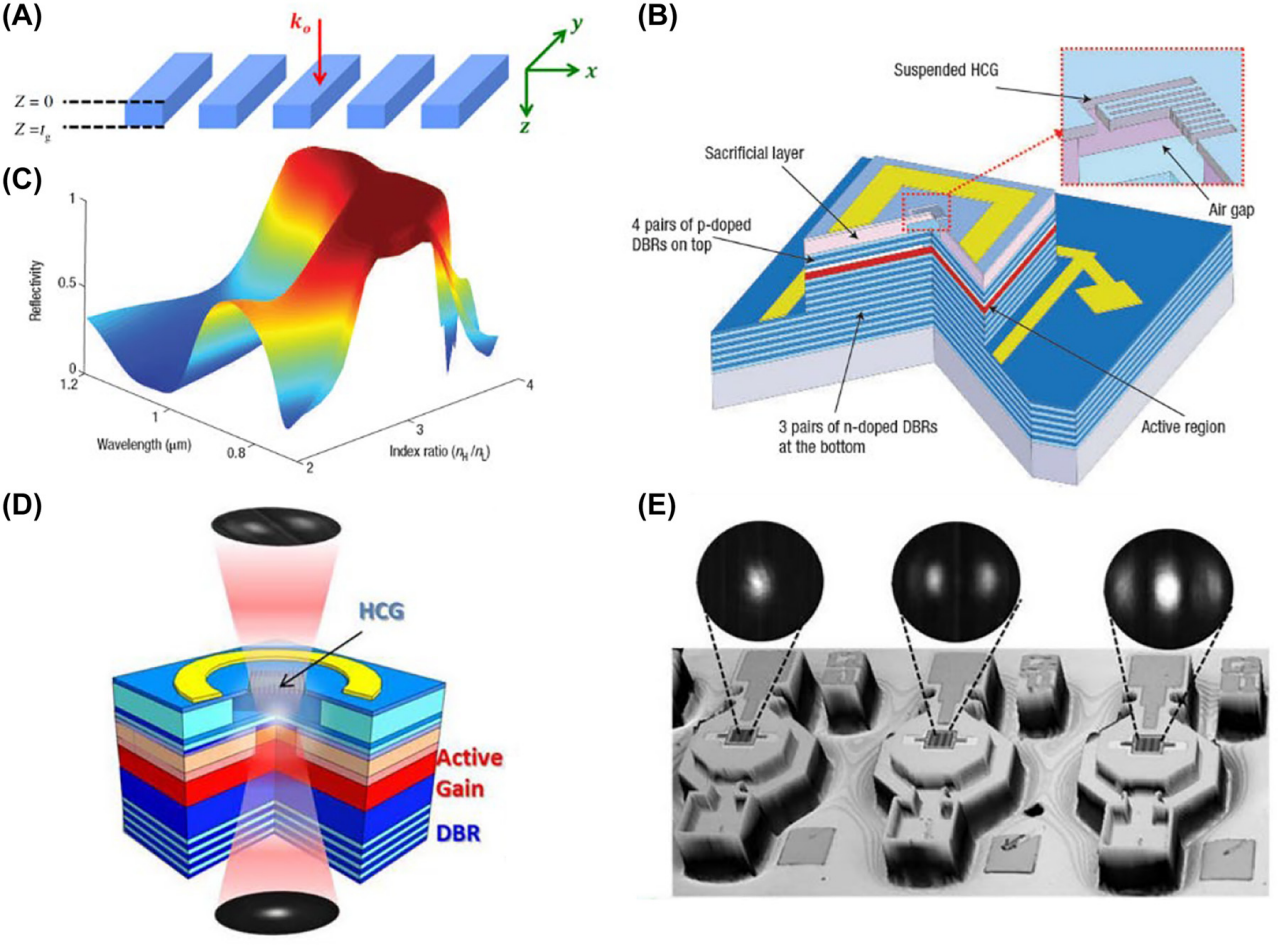
VCSEL with an HCG reflective mirror. (A) Schematic of the HCG. (B) Schematic cross-sectional view of the VCSEL with suspended HCG mirror. (C) Simulated reflectivity of the TM light versus the wavelengths and *n*_H_/*n*_L_ (the index ratio between the high and low refractive index materials). (D) VCSEL with HCG mirror for beam shaping. Two beams emitted from the top and bottom have different intensity profiles. (E) Three VCSELs with varying designs of HCG are designed to generate different intensity profiles. Device images are taken with a 3D confocal optical microscope. Far-field patterns of the emitted beams are taken with an IR camera. From left to right: single-lobe, double-lobe and triple-lobe. Reprint permission obtained for references [66, 76, 77].

Gratings made of high refractive index materials, such as silicon, and surrounded by low refractive index materials are often referred to as high contrast gratings [66] (HCG). Each grating bar can be considered as a waveguide along the *z*-direction (Figure 4A). The incident light couples into the waveguide modes, propagates back and forth within the waveguide and leaks energy in the incident plane (*z* = 0) and exiting plane (*z* = *t*_g_) at each round trip. Thus, the phase and amplitude of transmitted/reflected light are determined by the geometrical parameters of the grating. For instance, to achieve high reflection, the HCG parameters should be chosen to yield destructive interference at the exiting plane so that the transmission is zero (or close to it) [67]. Similarly, an HCG can be designed to realize beam steering [68–70], focusing [71], structural colour generation [72], and other functions [73–75].

HCGs with high reflectance and broad bandwidth have been used to replace the DBRs of VCSELs to reduce the footprint, solve the small DBR bandwidth problem and provide polarization mode selection [76, 78]. The VCSEL of Figure 4B has a conventional n-type DBR mirror at the bottom, an active region, four pairs of top p-type DBRs and a suspended HCG. To fabricate the device, the grating pattern is first defined in the e-beam resist with EBL, then transformed to the Al_0.6_Ga_0.4_As layer by RIE. The sacrificial layer beneath the HCG is chemically etched away to release the HCG and make it free-standing, followed by a critical point drying process. The HCG is polarization-sensitive. Its reflectance for the TM-polarized light (electric field perpendicular to the grating grooves) is greater than 99.9% for the wavelength range 0.8–0.88 μm (Figure 4C) but is only 95% for the TE-polarized light. As a result, only the TM mode meets the lasing condition. The light-intensity characteristic of the device is measured for both TM and TE modes, and suggests that the suppression ratio can reach up to 20 dB. The HCG also helps with the single-mode emission due to its size-dependent reflectivity: High-order modes with large beam cross sections have different reflectivity compared to the fundamental mode due to the limited extent of the HCG (10 × 10 μm). The emission spectra of the device are measured under different injected currents, ranging from 1.0 to 3.0 mA. The results show that the suppression ratio of the high-order modes can be 45 dB. Huang et al. [79] also integrate the HCG with nanoscale actuators to create a movable mirror. This means that the cavity length of the VCSEL can be altered, and thereby the emission wavelength.

The ability to shape laser beams is beneficial because many applications require intensity distributions that differ from the typical Gaussian beam that results from a laser cavity [80]. For example, a beam with a flat top is advantageous for material processing because it allows cuts to be made with vertical sidewalls. Recently, Li et al. [77] reported a VCSEL device with a high-contrast metasurface mirror as a beam-shaping element (Figure 4D). The HCG is fabricated in the InP layer on the emitting surface [81, 82]. The HCG transmittance is highly dependent on the incident angle, with its geometrical parameters determine the incident angle-transmittance relation. The authors show that if the HCG transmission is lower for the center than on the sides, the emitted light (at 1550 nm) has a double-lobe far field pattern rather than a Gaussian (Figure 4E). A two-faced VCSEL, which emits a shaped beam from the top HCG and Gaussian shaped beam from the bottom DBR, is also experimentally demonstrated.

## 3 VCSELs with waveguides

Another approach to the monolithic integration of metasurfaces with semiconductor lasers is to make use of a dielectric or plasmonic waveguide to link them. In the dielectric waveguide approach, the emitted light couples into one end of the waveguide and propagates to the other end, where it is transformed by the metasurface. In the plasmonic approach, the emitted light is coupled into SP waves that are then manipulated by the integrated plasmonic circuitry. These systems have outstanding potential for applications such as communications and lab-on-a-chip devices.

**Figure 5:**
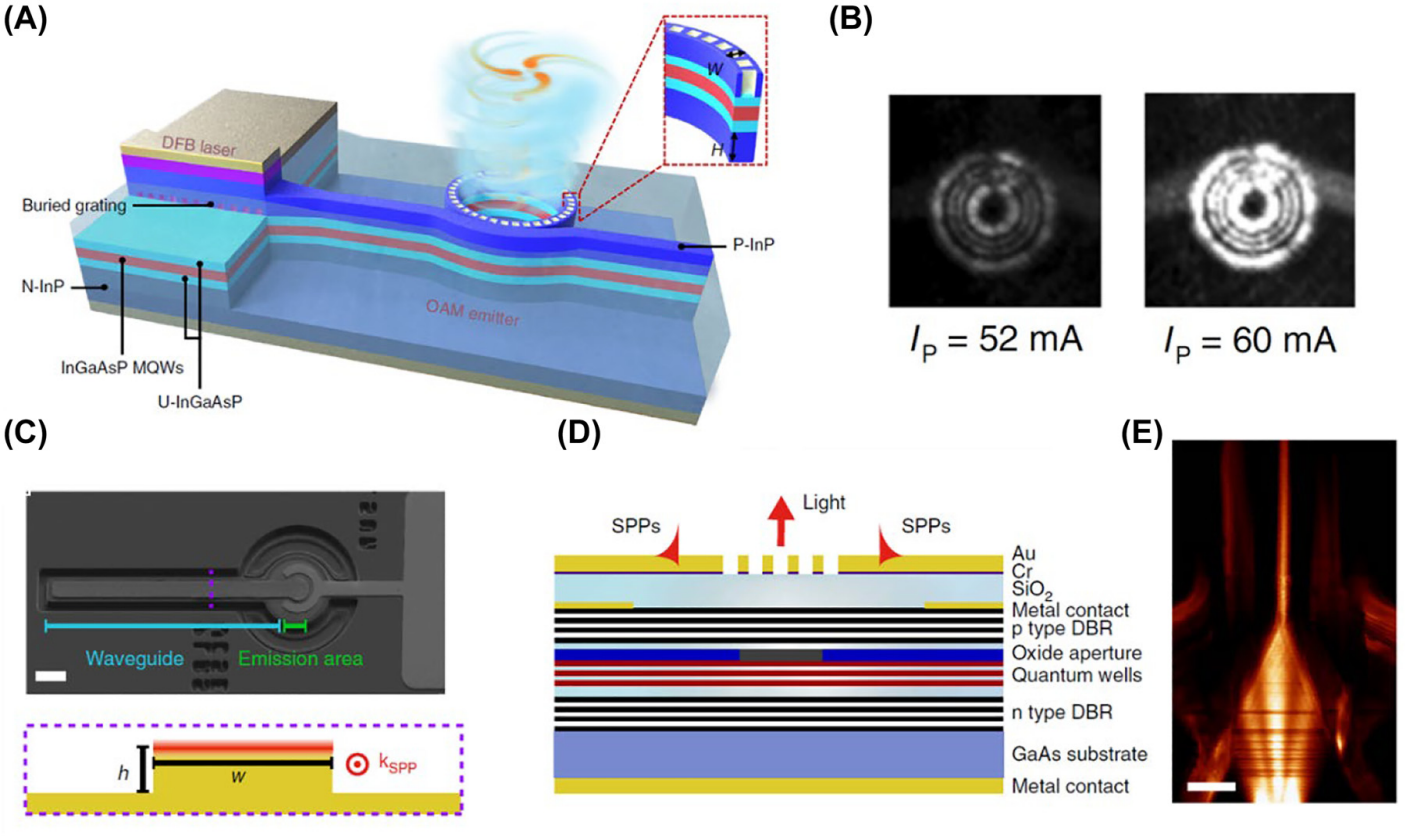
VCSELs with waveguide structures. (A) Schematic of DFB laser with ridge waveguide and ring-shaped OAM emitter. (B) Experimentally-obtained near-field pattern of emitted light from OAM emitter. *I*_p_ represents the pumping current. (C) Top: SEM image of VCSEL with a plasmonic waveguide. Scalebar: 20 μm. Bottom: schematic of waveguide cross-section. *k*_spp_ denotes the wavevector of the SPP. (D) Schematic cross-section view of the VCSEL with a plasmonic waveguide. SPs are excited by the emission area’s grating structure and propagate along the Au/air interface. (E) Experimentally-obtained near-field intensity distributions of single-mode plasmonic waveguide. Scalebar: 5.4 μm. Reprint permission obtained for references [83, 84].

Zhang et al. [83] proposed a method to link a DFB laser with a micro-ring-based optical vortex emitter via a dielectric waveguide structure (Figure 5A). The device consists of three parts: a DFB laser located in the buried grating area, a ridge waveguide and a ring-shaped OAM emitter. Device fabrication includes the following. The ohmic contact layer and part of the top InP cladding layer on the right side of the DFB laser are removed to reduce the absorption and scattering of the top hole-grating (on the ring-shaped OAM emitter). The mesa-structure of the DFB laser, the ridge waveguide and OAM emitter with hole-gratings can be fabricated in a single dry-etching step. The etching process uses a SiN_*x*_ mask and a mixture of CH_4_ and H_2_. A feature of the dry etching process is that the etch rate depends on the mask geometry. As a result, the shallow hole-grating and the deep ridge can be simultaneously formed in one etch step by appropriate choice of the etching parameters. When the light propagates along the waveguide, it couples unidirectionally into the ring OAM-emitter. If the light is on-resonance within the ring, the scattered light will skew in the azimuthal direction and transform into a helix based on Huygens’ Principle [85]. Thus, an OAM beam will be emitted vertically.

McPolin et al. [84] integrated plasmonic structures on a VCSEL for SPP manipulation and detection. As shown in Figure 5C, the device consists of an emission area (VCSEL) and a waveguide. The mesa structure of the VCSEL and the extended ridge beneath the plasmonic waveguide are formed simultaneously by lithography and dry etching. Selective oxidation forms the current limiting aperture in the VCSEL mesa and isolates current in the extended ridge. The p-and n-type metal contacts of the VCSEL are then deposited, and the lift-off process forms the Au-Cr plasmonic waveguide. By appropriate design of the SPP grating couplers and waveguides, SPP excitation, guiding, frequency conversion and detection are experimentally verified on this platform (Figure 5D–E).

## 4 External cavity semiconductor lasers with metasurfaces

Due to the compactness of semiconductor lasers, it is usually challenging to incorporate metasurfaces within the laser cavity. The external cavity configuration provides a feasible way to control the lasing mode in the cavity. Commercially available semiconductor lasers can be used, and the metasurface integration process can be avoided. Although we focus on metasurfaces in this subsection, it is worth mentioning that researchers have used other optical elements in the external cavity configuration. For example, spatial light modulators have provided feedback to the cavity to generate OAM modes directly [88]. Two-dimensional gratings and birefringent crystals were also used in external cavities to generate Airy beams [89] and vector beams [90], respectively.

**Figure 6:**
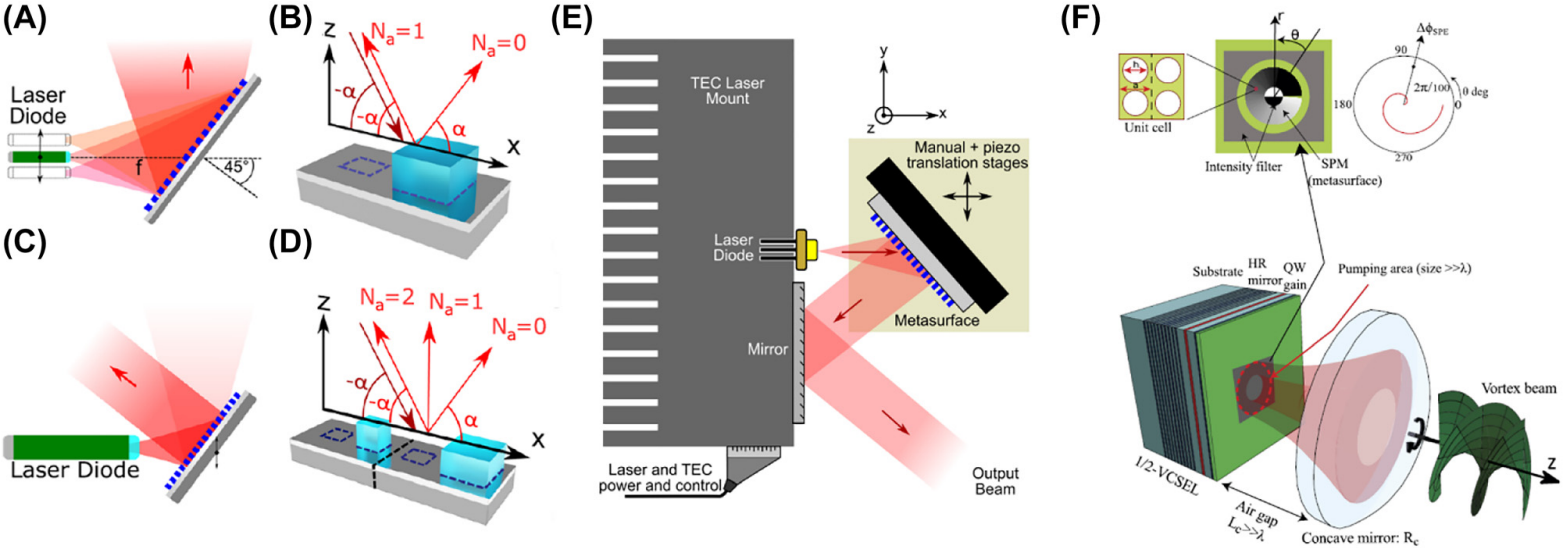
External cavity semiconductor lasers with metasurfaces. (A) Schematic of an external cavity laser diode with reflective metasurface for output wavelength tuning. (B) Schematic of a supercell containing one nanopillar. *N*_a_ represents the order of diffraction. The incident light is opposite to the direction of the first order. (C) Schematic of an external cavity laser diode with reflective metasurface for output collimation. (D) Schematic of a new supercell formed by two supercells in (B). The incident light is opposite to the direction of the second order. (E) Experimental setup to characterize the metasurface-based external cavity laser diode. The laser diode is mounted on a thermoelectric cooling mount. The metasurface is placed on a three-axis stage, where its position is tuned by manual micrometer screws and piezoelectric translation. The light is reflected away from the mount by the metasurface and a mirror. Spectrum of the output beam is measured with a fiber-coupled grating spectrometer. Holographic image formed by the output beam is captured with an imaging system. (F) Bottom: schematic of the external cavity vortex laser with metasurface on the emitting facet. Top: unit cell of the metasurface, schematic of the spiral phase metasurface (SPM) and the intensity filter, azimuthal phase variation Δ*ϕ*_SPM_ introduced by the metasurface (from left to right). Reprint permission obtained from references [86, 87].

Recently, Spägele et al. [86] demonstrated reflective metasurface-based external cavity lasers that are able to tune the wavelength and shape the wavefront of output beams. As shown in Figure 6A, the laser diode (LD) emits a beam with large divergence and illuminates the metasurface consisting of TiO_2_ nanopillars on a silver substrate. The metasurface splits the s-polarized light into two diffraction orders (Figure 6B). The zeroth order is the output beam and is specular reflected. The first order is focused back into the LD to provide feedback for the cavity. Due to the tilt of the metasurface and the chromatic aberration, the position of the focal point (along the vertical direction) in front of the LD changes with the wavelength [91, 92]. So, if the position of the LD is tuned in the vertical direction, the wavelength of the light that is efficiently focused back into the cavity will be change, thereby determining the lasing wavelength. The wavefront of the output beam can be controlled by designing the supercell structure that simultaneously meets the complex amplitude requirements of the relevant orders (Figure 6C–D). For instance, the output beam can be collimated, or shaped into an arbitrary holographic image. The experimental setup used to characterize the device is shown in Figure 6E. While the metasurface is not integrated with the VCSEL, we nonetheless include the work in this Review as we feel it may prompt future work on metasurface integration.

Seghilani et al. [87] fabricated a dielectric metasurface consisting of holes of varying diameters in a Si_3_N_4_ film on the emitting surface of a VCSEL (Figure 6F). The Si_3_N_4_ film is 125 nm thick, and the holes are fabricated using e-beam lithography and RIE etching. The hole diameter determines the effective refractive index of the metasurface, which introduce a weak azimuthal phase variation Δ*ϕ*_SPM_ for the modes in the VCSEL. The phase profile of two OAM beams of opposite topological charges will thus be perturbed, but to different levels. As a result, one of the OAM beams will be selected by mode competition. A Mach–Zehnder interferometer is used to experimentally characterize the device. A collimated vortex beam in one arm interferes with a copy of itself having a curved wavefront in another arm. The curved wavefront is achieved by inserting a lens with a short focal length in the arm. The setup can not only verify the topological charge of the generated OAM, but also detect its handedness.

**Figure 7:**
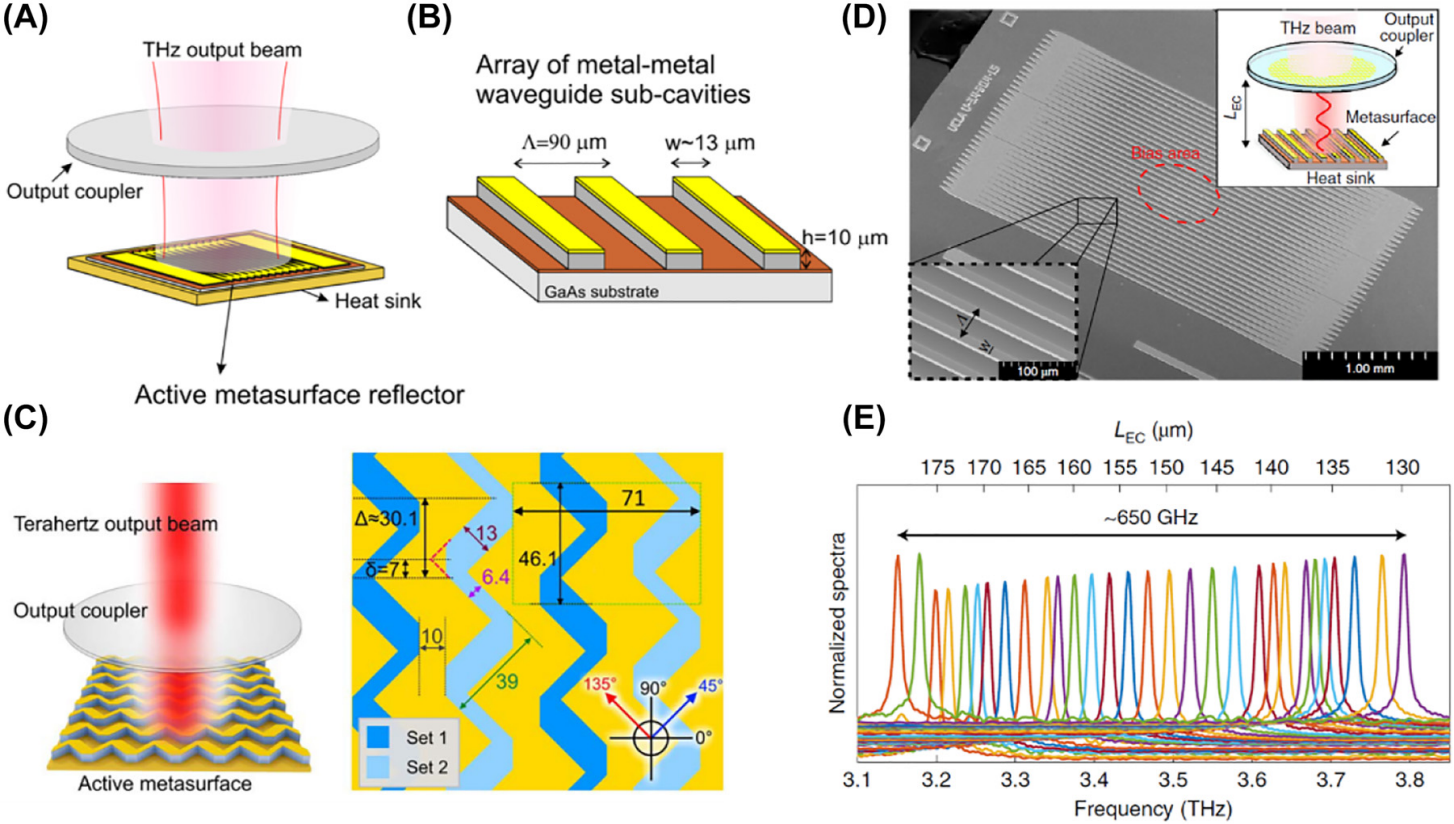
External cavity surface emitting QCL with metal-metal type active metasurface. (A) Schematic of a THz external-cavity QCL. (B) Schematic of the metal-metal waveguide type metasurface. (C) Schematic of the polarization switchable device (left) and the zig-zag type metasurface (right). (D) SEM image and schematic of the wavelength-tunable external-cavity QCL. (E) Spectra of the output beams when tuning the length of the cavity *L*_EC_. Reprint permission obtained from references [93–95].

Xu et al. [93] proposed an external-cavity surface emitting quantum cascade laser in the terahertz range. The device contains an active metasurface reflector above the heat sink and an output coupler on the top (Figure 7A). A schematic of the metasurface reflector is shown in Figure 7B. It consists of an array of metal-metal waveguides. Each waveguide is a sandwich structure containing a quantum well semiconductor active material between the metal layers. The fact that the modes of the waveguide are confined to the active material is beneficial for heat removal, allowing continuous wave operation. To make the metasurface reflector, a GaAs substrate (device wafer) containing the active material (10 μm thick) is bonded to a receptor wafer through Cu–Cu thermocompression bonding [96, 97]. Both wafers are coated with Cu on the bonding side. This is followed by removal of the GaAs substrate of the device wafer by wet etching. Next, three thin metallic layers, Cr, Au and Ni are deposited on the active material and lift off is performed, yielding the top metallization and etching masks for the waveguide ridges. The waveguide ridges are defined by RIE. This is followed by removal of the top Ni layer. A near-Gaussian beam with >5 mW output power is realized. Since the light emitted from the metasurface is perpendicular to the quantum-wells, an output coupler with polarization selectivity can be used to optimize the output power and efficiency.

A further refinement of this approach is as follows. As shown in Figure 7C, a metasurface is formed by interleaving two sets of zig-zag antennas for use in the external-cavity QCL [94]. The antennas have metal-metal structures similar to those of Figure 7B. The zig-zag antennas contain patches with widths of 13 μm. Each patch has similar structure with the grating ridges shown in Figure 7B, which contains an Au/Ti layer on the gain materials. These patches interact with light of polarized across the patches. The patches with widths of 6.4 μm are only used to link the functional patches. As a result, the antennas Set 1 and Set 2 interact with 45° and 135° polarized light, respectively. If we bias the two sets separately, polarization of the output beam can be dynamically switched. The authors furthermore demonstrated that a piezoelectric stepper motor could be used to control the position of the output coupler, thereby tuning the device’s cavity length and hence the lasing wavelength [95] (Figure 7D–E).

## 5 Conclusions and outlook

Semiconductor lasers integrated with metasurfaces combine the advantages of both to form compact devices with versatile functionalities. This has been enabled by progress in design principles and nanofabrication. The resultant devices operate from the visible to the THz, and are an enabling technology for industrial use, consumer electronics and scientific research. As an example of what is possible, consider the work of Xie et al., who decorated each VCSEL of a VCSEL array with a beam-steering metasurface [41]. Each metasurface was chosen to have a different steering angle, enabling the realization of wavefront scanning by turning on the appropriate VCSEL of the array. Semiconductor lasers with Dammann-grating type metasurface [98] can directly project a 2D beam array, which is desirable for facial recognition and angular sensing applications. If integrated with high numerical aperture metalenses [99], semiconductor lasers can have numerous scientific applications in optical trapping, laser-based microscopy, and so on. In addition to the wavefront control, the integrated devices can be used for polarization and wavelength control as well, which may benefit the fields like optical communications and optical sensing.

In this paper we focus on semiconductor lasers as a platform for metasurfaces integration, but the design principles and fabrication techniques could be applied to many other optical devices. For instance, geometric metasurfaces can be inserted in the cavity [100] or work as a cavity mirror [101, 102] of a solid-state laser for OAM modes generation. Lasing in hybrid lattice resonance metasurfaces [103] and quasi-periodic/aperiodic Ag lattices [104] have been demonstrated. Metasurfaces can also help with other light sources, such as quantum dots [105], X-ray sources [106], light emitting diodes [107–110], and quantum emitters [111]. In addition to the light sources, the integration of metasurfaces with waveguides [112–114] and photodetectors [115–117] enable unusual functionalities in a platform that is inherently miniaturized.

Despite the successful demonstration of metasurfaces-integrated devices, important challenges remain. First, semiconductor lasers are a mature technology and produced in large volumes with excellent uniformity and low cost. This is not the case for metasurfaces, so in some sense they represent the weak link of the integrated device. Metasurfaces are generally fabricated by e-beam lithography or focused ion beam, which are impractical for large-scale applications. If we compare commercially available optical elements, such as achromatic lenses and beam shapers, with their metasurface counterparts, the latter do not fare as well in terms of transmission efficiency and size. This hinders their large-scale applications. Second, the integration processes are usually complicated and not compatible with the industry-standard methods for semiconductor laser fabrication. It is encouraging to see that many attempts are being made to overcome the problems of metasurface cost [118–120] and quality [121, 122] (efficiency, tunability, etc.). In order to solve the problems in the integration process, researchers are trying to incorporate metasurfaces into structures such as light emitting diodes [123]. We can also learn from the solutions that have been employed in silicon photonics [124]: Due to the lack of an integrated efficient, reliable and electrically-pumped silicon laser, III–V materials-based semiconductor laser chips are separately processed and flip-chip integrated on the silicon circuits. In another approach, to make the fabrication process of III–V material lasers compatible with CMOS, researchers are developing wafer-bonding techniques or are performing direct epitaxy of III–V quantum dots on silicon. Inspired by these rapidly developing techniques, perhaps we can flip-chip bond large-scale fabricated metasurface chips onto semiconductor laser arrays. The wafer-bonding and direct epitaxy of quantum dots on metasurface are also possible paths that can be explored. Due to the compelling applications and the current rate of progress, we envisage that metasurface-integrated optical elements will play critical roles in future optoelectronic systems.
